# Direct and moderating effects of personality on stigma towards mental illness

**DOI:** 10.1186/s12888-018-1932-3

**Published:** 2018-11-06

**Authors:** Qi Yuan, Esmond Seow, Edimansyah Abdin, Boon Yiang Chua, Hui Lin Ong, Ellaisha Samari, Siow Ann Chong, Mythily Subramaniam

**Affiliations:** Research Division, Institute of Mental Health, Singapore, Singapore

**Keywords:** Personality, Stigma, Mental illness, Interpersonal contact, Moderation

## Abstract

**Background:**

While many studies have explored the concept and correlates of stigma towards individuals with mental illness, few have investigated the role of personality in this process. In the current study, we firstly examined the relationship between personality and stigma towards mental illness; and then explored the moderating effects of personality traits on the relationship between contact experience/s and stigma.

**Methods:**

Participants were recruited from public medical (*N* = 502) and nursing schools (*N* = 500) from April to September 2016 in Singapore for this cross-sectional survey, and they were randomly assigned to a vignette describing one of the following mental disorders: major depressive disorder, obsessive compulsive disorder, alcohol abuse, schizophrenia, and dementia. Stigma was measured by the ‘Personal and Perceived scales of the Depression Stigma Scale’ and the ‘Social Distance Scale’. These scales together had a 3-factor structure based on a previous national study in Singapore, namely ‘weak-not-sick’, ‘dangerous/unpredictable’ and ‘social distance’. Personality was measured by the 20-item short form of the International Personality Item Pool-five factor model measure.

**Results:**

Regression suggested agreeableness and openness to experience were negatively associated with all three domains of stigma. ‘Weak-not-sick’ and extraversion were positively associated; and ‘social distance’ was positively associated with higher scores on conscientiousness and neuroticism. Both close- and non-close contact were associated with more positive attitudes towards mental illness among the participants. Openness to experience moderated the relationships of close contact experience with ‘weak-not-sick’ and ‘dangerous/unpredictable’, but in different directions. The association between close contact and ‘social distance’ were moderated by agreeableness.

**Conclusions:**

Unlike non-close contact experience, close contact with people with mental illness worked differently on stigma for individuals with different personality traits. Future studies are needed to further explore the underlying mechanisms for such differences.

**Electronic supplementary material:**

The online version of this article (10.1186/s12888-018-1932-3) contains supplementary material, which is available to authorized users.

## Background

The World Health Organization defined stigma as ‘a distinguishing mark establishing a demarcation between the stigmatized person and others attributing negative characteristics to this person’ [1]. Stigma towards mental illness is widespread, and comprises public stigma (the reaction that the general population has to people with mental illness) and self-stigma (the prejudice which people with mental illness turn against themselves) [2]. It has three components- stereotypes (negative belief about a group), prejudice (negative emotional reaction) and discrimination (behavioral response to prejudice) [2, 3]. For people with mental illness, self-stigma could affect their personal experience with others, their willingness to disclose their disease, and the subsequent help-seeking behavior [3, 4]; while for public stigma, it could affect the daily interactions between the public and the mentally ill [3, 5]. Both have the potential to lead to negative outcomes among individuals with mental disorders. Other than the general public [6, 7], health care professionals may also hold stereotypes, and have negative emotional reactions and behavioral responses towards people with mental illness [8, 9]. Such stigma can be detrimental with regards to the quality of health care received by the mentally ill [8]. A previous study among health care providers in the United States found that professionals who endorsed stigmatizing characteristics of patients with mental illness were more likely to view the patients as non-adherent, making them less likely to refer patients to specialists to refill medications [10]. Findings from a previous systematic review also suggested that while delivering healthcare services to patients with substance abuse disorders, healthcare professionals tended to use a more avoidant approach compared to those without such disorders, which in turn could influence the treatment outcomes [11]. Another study among family physicians in United States showed that the physicians tended to believe that the physical symptoms (e.g. headache and abdominal pain) among people with previous episodes of depression stemmed from their mental disorders and as a result they were less likely to investigate the underlying reasons [12]. To lower the negative impact on patients, it is necessary to improve the attitudes of healthcare professionals towards individuals with mental illness, especially among those with relatively high stigma. Thus, identifying factors that influence stigma could be one of the crucial steps in this endeavor.

Various studies have explored relationships between personality and prejudice, mainly from two theoretical perspectives 1) the authoritarian personality approach which proposed a direct link between personality and prejudice, and 2) a dual-process motivational approach which focused on personalities’ indirect impact over prejudice [13]. For example, Ekehammar and Akrami [14, 15] tested the relationships between the Big Five personality traits and generalized prejudice (a composite measure based on scores from racial/ethnic prejudice, attitudes towards women, and attitudes towards mentally disabled people, and attitudes towards homosexuals, lesbians and gay men) among two different samples of students, and found consistent significant negative relationships of openness and agreeableness with generalized prejudice. Their findings are consistent with that from a meta-analysis exploring the relationship between personality and prejudice [13]. On the other hand, a 2008 review of 18 different models suggested that although prejudice and stigma have different emphasis and focus (i.e. subjects), their conceptual models including the cause and consequences actually have considerable overlap [16]. And the authors also suggested that this large degree of overlap should encourage scholars to reach across stigma/prejudice lines while searching for theory, methods and empirical findings [16]. As a result, findings on relationships between personality and prejudice might be extended to between personality and stigma (i.e. stigma is not always related to lack of knowledge [17], other factors including personality might also play an important role). Preliminary evidence supports this extension - according to a study conducted by Arikan among 700 final year university students, a strong correlation between the use of narcissistic defense (i.e. narcissistic personality) and the tendency to stigmatize people was found [18]. Another study exploring the social appraisal of adults with attention deficit hyperactivity disorder (ADHD) among psychology undergraduates suggested that agreeableness, extraversion, and conscientiousness were all significantly associated with their attitudes towards people with ADHD [19]. More specifically, Brown [20] also found that after controlling for experiences of personal contact with people with mental illness, openness and agreeableness were negatively associated with stigma towards severe mental illness. However, till date studies on this topic are very limited; and none of them are among Asian populations.

Inter-group contact experience is beneficial for reducing prejudice [21]; and a similar relationship also applies for contact and attitudes to mental illness [22, 23]. One potential explanation is that contact experiences help people to understand the feelings and worldview of the stigmatized group and as a result lead to enhanced empathy towards the stigmatized groups and reduce prejudice [24]. Previous studies on personality and communication skills suggested that agreeableness and openness to experience uniquely predicted better active-empathic listening skills of individuals [25]. For those scoring higher on openness, their state of being more open-mind [26, 27] might allow them to gain more understanding on the feelings and worldview of the individuals with mental illness from the contact experience. People who score higher on agreeableness tend to be more good-natured, cooperative and tolerant [26, 27]; this in turn might enable them to have enhanced empathy towards people with mental illness. Following this rationale, we hypothesized that higher levels of these two personality traits might contribute to a more positive inter-group contact process, and ultimately lead to larger improvements on stigma towards mental illness compared to those who score lower on these traits. In other words, other than the direct relationship, personality traits including openness and agreeableness might also work as moderators which moderate the relationship between contact experience/s and stigma towards mental illness.

The current study aims to 1) examine the direct relationship between the ‘Big Five’ personality traits and stigma towards mental illness through a sample of health care students in Singapore; and 2) explore if agreeableness and openness to experience moderate the relationship between contact experience and stigma towards mental illness.

## Methods

### Participants and procedure

Data for the current study were extracted from a project exploring mental health literacy level of health care students using a vignette-based approach as well as factors that lead them to choose psychiatry as a career [28, 29]. This cross-sectional study used an online web survey, QuestionPro®, to collect data. The target population included medical and nursing students who were enrolled in public medical and nursing educational programmes in Singapore at the time of the study from April to September 2016. Students who were Singapore residents (Singapore Citizens or Permanent Residents) were eligible for the study.

Public medical and nursing schools in Singapore were approached by the study team members to seek their permission to recruit respondents from these institutions. Upon approval, the institutions sent a mass email on behalf of the study team to invite the students to join the study, with a pre-defined quota for each institution. The quota was based on institutions, the academic years and also the number of vignettes. This was meant to improve the representativeness of the study sample. The participation was voluntary, and an online consent form was used to obtain the informed consent from all participants. The online survey took around 20–30 min to complete; and after completion, a SG$20 voucher was given to each participant. In total, 1002 students were recruited during the recruitment period, among which 502 were medical students and 500 were nursing students.

Ethical approval of the project was granted by the National Healthcare Group Domain Specific Review Board in Singapore.

### Measurements

#### Personality traits

Personality was measured by the 20-item short form [30] of the 50-item International Personality Item Pool-Five-Factor Model measure (mini-IPIP) [31], with 4 items measuring each of the ‘Big Five’ personality traits (i.e. extraversion, agreeableness, conscientiousness, neuroticism, and openness to experience). Participants were required to read items such as ‘I am the life of the party’, ‘I sympathize with others’ feelings’, and then rate how each item accurately describe themselves using a 5-point Likert scale, with ‘1 = very inaccurate’ and ‘5 = very accurate’. An average score was calculated for each personality trait; with a higher score representing higher endorsement of the personality trait. The composite reliability statistics were 0.79 for extraversion, 0.79 for agreeableness, 0.68 for conscientiousness, 0.71 for neuroticism, and 0.76 for openness in the current study. A cut-off of 0.7 for composite reliability [32] was deemed acceptable.

#### Stigma towards mental illness

Participants of the current study were randomly assigned to a vignette describing one of the following specific mental disorders: major depressive disorder, obsessive compulsive disorder (OCD), alcohol abuse, schizophrenia, and dementia; and were then asked to indicate their attitudes to the mentally ill person described in the assigned vignette using two different scales - the personal subscale of the ‘Personal and Perceived scales of the Depression Stigma Scale (DSS)’ [33] and the ‘Social Distance Scale (SDS’) [34]. Vignettes on depression and schizophrenia were adapted from those used in previous studies [35, 36]; while those on dementia, alcohol abuse and OCD were developed by the investigators [7, 37]. All these vignettes were approved by a panel of senior clinical psychiatrists to ensure that each disorder satisfied the DSM-IV diagnostic criteria. Please refer to Additional file 1 for the details of the vignettes.

The DSS has two subscales (personal stigma and perceived stigma), and each comprises 9 different items [33]. It was originally developed to measure depression stigma. In the current study, only 8 items of the DSS-personal subscale was used (item ‘I would not vote for a politician if I know they had a mental illness’ was excluded) [7]. The DSS was rated based on a 5-point Likert scale, ranging from ‘1 = strongly disagree’ to ‘5 = strongly agree’. The SDS has 5 different items, and it was used to measure the participants’ self-reported willingness to contact the person depicted in the vignette. A 4-point Likert scale varied from ‘1 = definitely unwilling’ to ‘4 = definitely willing’ was used for its rating. This vignette-based approach and the measurement tools have been used in a national study on mental health literacy in Singapore before, and the factor analysis suggested a 3-factor structure – ‘weak-not-sick’, ‘dangerous/unpredictable’, and ‘social distance’ [7]. Following the national study, the 5 items of ‘social distance’ were reversed scored first; and then the scores for all three factors were calculated by summing the items included in each factor (for ‘dangerous/unpredictable’, item ‘if I had a problem like [vignette] I would not tell anyone’ was excluded from the calculation) [7]. In this case, higher scores of each factor represent more stigmatizing attitudes towards mental illness. In the current sample, we ran a confirmatory factor analysis for this 3-factor structure, and the results suggested acceptable model fit (the comparative fit index/CFI = 0.960, the Tucker-Lewis index/TLI = 0.948, the root mean square error of approximation/RMSEA = 0.084). The composite reliability statistics were 0.73, 0.73, and 0.90 for ‘weak-not-sick’, ‘dangerous/unpredictable’ and ‘social distance’, respectively.

#### Contact experiences

Contact experiences were captured by two different questions after the participants read the assigned vignette: 1) close contact – ‘has anyone in your family or close circle of friends ever had problems depicted in the vignette?’; and 2) non-close contact – ‘have you ever had any experiences (e.g. volunteering, training) in dealing with a person who had problems depicted in the vignette?’. In the current study to ensure the interpretability, participants who reported ‘don’t know/refused’ for these two contact experience questions were treated as missing in all regression analyses. The total sample included in the regression and path analyses was 870.

Other than the above-mentioned assessments, we also measured socio-demographics including age, gender, ethnicity, highest education level attained before current school, monthly household income, and type of study program (i.e. medical or nursing), vignette type and whether the participants could correctly identify the mental disorder described in the vignette assigned to them.

### Statistical analysis

Descriptive analysis was conducted for socio-demographics and other variables. Mean and standard deviation (SD) were calculated for continuous variables; while for categorical variables, their frequency and percentage were presented. To explore the direct effects of personality on stigma, linear regression analyses were conducted, with each factor of the stigma measures regressed on all 5 ‘Big Five’ personality traits, after controlling for socio-demographics (including age, gender, ethnicity, education level, monthly income, and type of study program), contact experiences (close contact and non-close contact), and vignette-associated variables (vignette type and the status whether the participants could correctly recognize the mental disorder in the assigned vignette). The regression was done through IBM SPSS V23.0, and a two-sided *p*-value below 0.05 was considered as statistically significant.

The moderating effects of agreeableness and openness to experience were tested through path analysis following a 2-step approach. In the first step, all three domains of stigma were regressed on the two types of contacts (i.e. close contact and non-close contact) simultaneously, with the two personalities of agreeableness and openness to experience being the moderators for each of the regression paths. In step 2 based on the results from the previous step; the non-significant moderation paths were removed. Both steps controlled for socio-demographics, the vignette-associated variables, and the remaining three personality traits. Model fit was defined as 1) the comparative fit index (CFI) > 0.95; 2) the Tucker-Lewis index (TLI) > 0.95; and the root mean square error of approximation (RMSEA) < 0.06 [38]. Finally, the regression parameters from the fit model were presented. Conditional effects (both unstandardized and standardized) between contact experience and stigma based on different level of moderators and the simple slopes for the moderation analyses were also presented. All moderation analyses were conducted using ‘lavaan’ package [39] in the R statistical program.

## Results

The socio-demographics of the study participants are presented in Table 1. The study sample comprised 1002 participants, among whom 500 were from nursing programs and 502 were medical students. The participants had a mean age of 21.3 years (SD = 3.3), and the majority of them were female (71.1%, *n* = 712) and Chinese (75.3%, *n* = 754). Among the participants, 75.4% (*n* = 755) correctly identified the mental disorder described in the assigned vignette. 25.1% of the participants (*n* = 252) reported that they had close friends or family members with problems similar to that depicted in the assigned vignette (i.e. close contact experience); while 33.7% (*n* = 338) mentioned that they used to deal with people had similar problems in the vignette (i.e. non-close contact experience).

**Table 1:** Socio-demographic characteristics of the sample (*n* = 1002)

Group	Frequency	Percentage
Age (mean, SD)	21.3	3.3
Gender		
Male	290	28.9
Female	712	71.1
Ethnicity		
Chinese	754	75.3
Malay	141	14.1
Indian or others	107	10.7
Highest education attained before current school		
Secondary or below	251	25.1
Technical education	114	11.4
A level	428	42.7
Diploma	81	8.1
Tertiary	128	12.8
Monthly household income		
Below 2000	241	24.1
2000-3999	263	26.3
4000-5999	171	17.1
6000-9999	146	14.6
More than 10,000	181	18.1
Type of study program		
Medical	502	50.1
Nursing	500	49.9
Vignette type		
Schizophrenia	200	20.0
Depression	200	20.0
OCD	201	20.1
Alcohol abuse	200	20.0
Dementia	201	20.1
Correct identification of vignette		
Yes	755	75.4
No	247	24.7
Close contact		
Yes	252	25.1
No	648	64.7
Don’t know/refuse	102	10.2
Non-close contact		
Yes	338	33.7
No	615	61.4
Don’t know/refuse	49	4.9

Table 2 presents the descriptive statistics for personality and stigma measures. The mean scores for each of the ‘Big Five’ personality traits are listed below: 2.91 for extraversion, 3.93 for agreeableness, 3.40 for conscientiousness, 2.86 for neuroticism, and 3.57 for openness to experience. For the three domains of stigma towards mental illness, the mean score was 7.90, 10.09, and 11.42 for ‘weak-not-sick’, ‘dangerous/unpredictable’, and ‘social distance’, respectively.

**Table 2:** Descriptive statistics for ‘Big Five’ personality and stigma measures

	Mean	SD	Min	Max
Big Five Personality				
Extraversion	2.91	0.85	1	5
Agreeableness	3.93	0.60	1	5
Conscientiousness	3.40	0.75	1	5
Neuroticism	2.86	0.76	1	5
Openness to experience	3.57	0.73	1	5
Stigma				
Weak-not-sick	7.90	2.37	3	15
Dangerous/unpredictable	10.09	2.71	4	19
Social distance	11.42	2.69	5	20

The linear regression results after controlling for covariates suggested that the ‘weak-not-sick’ factor was significantly associated with extraversion (*B* = 0.429, *p* < 0.001), agreeableness (*B* = − 0.350, *p* = 0.004), and openness to experience (*B* = − 0.340, *p* = 0.001). Significant associations were also identified for ‘dangerous/unpredictable’ factor with agreeableness (*B* = − 0.467, *p* = 0.002) and openness to experience (*B* = − 0.421, *p* = 0.001); and for ‘social distance’ with agreeableness (*B* = − 0.619, *p* < 0.001), conscientiousness (*B* = 0.251, *p* = 0.028), neuroticism (*B* = 0.238, *p* = 0.040) and openness to experience (*B* = − 0.419, *p* = 0.001). Meanwhile, having close-contact and non-close contact with individuals with mental illness were both significantly correlated with more positive attitudes on the three domains of stigma towards mental illness. Details of the results are shown in Table 3.

**Table 3:** Linear regression results of the direct relationships between personality and stigma (n = 870)

	Weak-not-sick					Dangerous/unpredictable					Social distance				
	*B*	β	95% CI		*p*	*B*	β	95% CI		*p*	*B*	β	95% CI		*p*
Extraversion	0.429	0.157	0.268	0.590	0.000	0.146	0.046	−0.056	0.347	0.157	0.035	0.011	−0.168	0.239	0.733
Agreeableness	−0.350	−0.088	− 0.587	− 0.113	0.004	−0.467	− 0.102	−0.764	− 0.170	0.002	− 0.619	− 0.137	− 0.918	− 0.319	0.000
Conscientiousness	0.081	0.026	−0.096	0.259	0.370	0.059	0.016	−0.163	0.282	0.600	0.251	0.070	0.026	0.475	0.028
Neuroticism	−0.101	−0.033	−0.281	0.078	0.269	0.152	0.043	−0.073	0.377	0.185	0.238	0.068	0.011	0.465	0.040
Openness	−0.340	−0.105	−0.534	− 0.146	0.001	− 0.421	−0.112	− 0.664	−0.178	0.001	−0.419	− 0.113	−0.664	− 0.174	0.001

^a^*B* – unstandardized coefficient;

^b^β – standardized coefficient;

^c^Controlled for socio-demographics (i.e. age, gender, ethnicity, education level, monthly income, and type of study) + contact experiences (i.e. close contact and non-close contact) + vignette-associated variables (i.e. vignette type and the status whether the participants could correctly recognize the mental disorder in the assigned vignette);

^d^Close contact was significant in predicting ‘dangerous/unpredictable’ (*B* = − 0.439, *p* = 0.032), and ‘social distance’ (*B* = − 0.800, *p* < 0.001);

^e^Non-close contact was significant in predicting ‘weak-not-sick’ (*B* = − 0.495, *p* = 0.001), ‘dangerous/unpredictable’ (*B* = − 0.369, *p* = 0.041), and ‘social distance’ (*B* = − 0.539, *p* = 0.003);

^f^Correct identification of the mental disorder in the vignette was significant in predicting ‘weak-not-sick’ (*B* = − 0.372, *p* = 0.028), ‘dangerous/unpredictable’ (*B* = 0.460, *p* = 0.031), and ‘social distance’ (*B* = 0.510, *p* = 0.018)

**Fig. 1:**
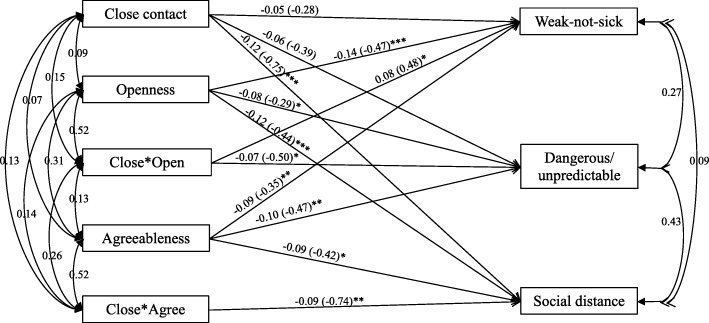
Final path model for the moderation analysis (*n* = 870). * *p* < 0.05; ** *p* < 0.01; *** *p* < 0.001. Close*Open: interaction between close contact and openness; Close*Agree: interaction between close contact and agreeableness. Moderators were mean-centered. Estimates are standardized path coefficients (unstandardized coefficients in the brackets to show the effects)

The path analysis suggested that after removing the insignificant moderation paths, the final model (Fig. 1) showed very good fit (χ^2^_(df)_ = 3.354_(3)_, CFI = 1.000, TLI = 0.990, RMSEA = 0.012). The final moderation model revealed that openness to experience moderated the relationships between close contact and ‘weak-not-sick’ (*B* = 0.48, z-value = 2.383, *p* = 0.017; interaction: Δ*R*^*2*^ = 0.0040), and between close contact and ‘dangerous/unpredictable’ (*B* = − 0.50, z-value = − 2.151, *p* = 0.031; interaction: Δ*R*^*2*^ = 0.0038); while agreeableness moderated the relationship between close contact and ‘social distance’ (*B* = − 0.74, z-value = − 2.631, *p* = 0.009; interaction: Δ*R*^*2*^ = 0.0051).

**Fig. 2:**
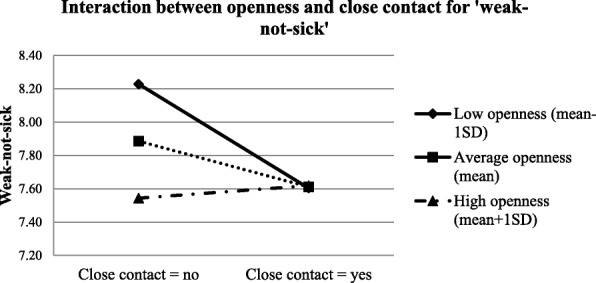
Interaction between openness and close contact for ‘weak-not-sick’

**Fig. 3:**
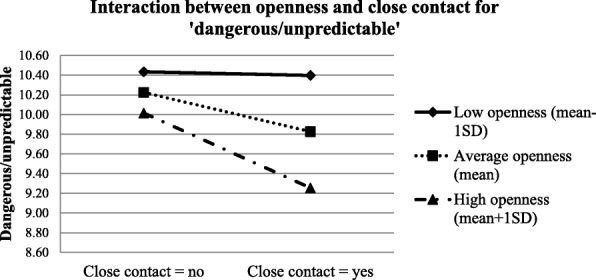
Interaction between openness and close contact for ‘dangerous/unpredictable’

**Fig. 4:**
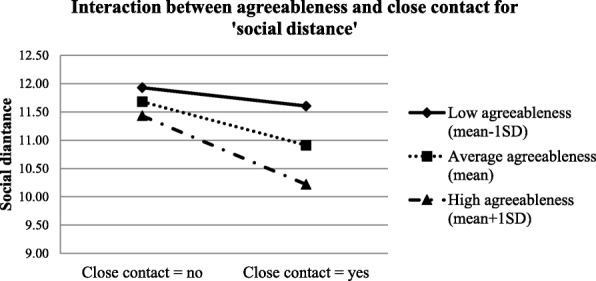
Interaction between agreeableness and close contact for ‘social distance’

Table 4 shows the results of the conditional effects of close contact experience on the three factors of the stigma measurements among the health care students at a specific level of openness to experience/agreeableness. Results suggest that at low openness to experience level (mean-1SD), ‘weakness-not-sick’ was negatively associated with close contact experience (conditional effect = − 0.625, *p* = 0.004); however, at average (mean) or high levels of openness to experience (mean + 1SD), the relationship was not significant. For ‘dangerous/unpredictable’, it was negatively associated with close contact experience only among participants with average (mean) or high openness to experience level (mean + 1SD). Close contact experience predicted less ‘social distance’ among people with average (mean) or high level of agreeableness (mean + 1SD). The graphic demonstrations of the moderations are shown in Figs 2, 3 and 4.

**Table 4:** Conditional effects of close contact on stigma at each level of personality

	Conditional Effect	SE	Standardized Conditional Effect	z-value	*p*
Weakness-not-sick					
Low openness (Mean-1SD)	−0.625	0.218	−0.129	−2.859	0.004
Average openness (Mean)	−0.275	0.161	−0.052	−1.701	0.089
High openness (Mean + 1SD)	0.075	0.192	0.025	0.393	0.695
Dangerous/unpredictable					
Low openness (Mean-1SD)	−0.037	0.263	0.003	−0.139	0.889
Average openness (Mean)	−0.398	0.202	−0.066	−1.974	0.048
High openness (Mean + 1SD)	−0.760	0.234	−0.135	−3.247	0.001
Social distance					
Low agreeableness (Mean-1SD)	−0.324	0.258	−0.043	−1.259	0.208
Average agreeableness (Mean)	−0.768	0.203	−0.129	−3.792	< 0.001
High agreeableness (Mean + 1SD)	−1.212	0.242	−0.215	−5.009	< 0.001

## Discussion

The current study aimed to explore the direct and moderating effects of personality on stigma towards mental illness using a vignette-based approach. Similar vignettes and measurement tools had been used in a previous national study in Singapore [7]. Compared to the young adults (aged 18 to 34 years) from the study by Subramaniam et al. [7], our health care students sample (mean age 21.3 years) reported lower scores on ‘weak-not-sick’ (7.90 vs. 9.58) and slightly lower scores on ‘dangerous/unpredictable’ (10.09 vs. 10.88); however, their scores on ‘social distance’ did not differ too much from the young people in the general population (11.42 vs. 11.58). Moreover, the multivariate regression results showed that ‘correct identification of the mental disorder depicted in the vignette’ was negatively associated with ‘weak-not-sick’, but positively associated with higher endorsement on ‘dangerous/unpredictable’ and ‘social distance’ towards people with mental illness. Both findings indicate that better knowledge on mental illness does not necessarily lead to lower discrimination (i.e. social distance), similar to findings from other studies [9, 17, 40].

The consistent negative relationships of agreeableness and openness to experience with stigma towards mental illness were confirmed by the multivariate regression analyses. This finding is consistent with results from the cross-sectional study on personality and stigma to mental illness conducted by Brown [20]. Studies have suggested that increases in level of empathy and perspective taking are helpful in reducing prejudice [24, 41], which might also be applicable for stigma. People scoring higher on agreeableness are generally more good-natured, cooperative and tolerant [26, 27], and as a result more likely to have higher empathy towards others, such as towards people with mental disorders. On the other hand, those who had higher scores on openness to experience are more open-minded [26, 27] and more likely to accept new ideas; which might ultimately contribute to a greater increase in perspective-taking towards the mentally ill among the participants. Another possible explanation could be relevant to social dominance orientation (SDO) and right-wing authoritarianism (RWA). Sibley and Duckitt [13] in their meta-analysis found that low agreeableness was associated with high SDO and low openness to experience was associated with high RWA, and both SDO and RWA were associated with more prejudice; moreover, the relationships between prejudice and agreeableness were fully mediated by SDO, while the relationships between prejudice and openness to experience were partially mediated by RWA. However, this alternative explanation assumes the similarities between prejudice and stigma [16, 42]. Since SDO and RWA were not measured in the current study, we were unable to confirm this effect in the current study.

Other than the above two personality traits, we also found significant positive associations between extraversion and ‘weak-not-sick’, between conscientiousness and ‘social distance’, and between neuroticism and ‘social distance’. Extraversion refers to being sociable, energetic or outgoing; while conscientiousness is about being efficient and organized [26]. According to research on prejudice, both extraversion [43] and conscientiousness [13, 43] were positively correlated with RWA; and high RWA is strongly correlated with prejudice and negative attitudes toward outgroups [43], such as individuals with mental illness in our case. However, since RWA was not measured in this study, future studies are needed to test whether RWA mediates the relationships between extraversion and conscientiousness with stigma. Previous studies on proxemics and neuroticism suggested that people who scored higher on neuroticism had a general tendency to prefer more social distance compared to those with lower scores [44].

The moderation analyses suggested that agreeableness and openness to experience do moderate the relationship between contact experience and stigma towards mental illness. However, unlike what we had expected, the moderating effect was limited only to the relationship between close contact and stigma. For non-close contact, the moderation was non-significant. To avoid over-interpretation, a sensitivity moderation analysis among the participants who were able to recognize the vignettes was also conducted and similar findings were identified. According to a previous literature review, contact experience of a more voluntary nature tended to be most effective in reducing negative attitudes towards people with severe mental illness [45]. Similar findings were also reported in studies on intergroup contact and prejudice, suggesting that the effect of intergroup contact experience on prejudice is not always positive; it might also lead to negative effects, especially when the contact involves involuntary contact [24, 46]. Compared to non-close contact, it is difficult for people to say ‘no’ to the close contact experiences, especially when the patients are family members of the participants. This difference, together with the individual differences in personality, might lead to the different results in the moderation analyses. This could be one of the potential explanations. Moreover, contact quality and valence might also contribute in this process. Barlow and colleagues [47] suggested that intergroup contacts could be positive and negative, and negative intergroup contacts could increase prejudice. In our study, since we did not gauge the differences in the quality of contact experiences, further studies are needed to explore this issue, and elucidate the exact underlying mechanism for this difference. We have also explored the potential moderation effects of the other three personality traits; however, none of them were significant.

Close contact experiences predicted significantly more positive attitudes on ‘dangerous/unpredictable’ among people with average or high openness to experience, and on ‘social distance’ among people with average or high agreeableness. However, the correlations were non-significant among those with low openness or agreeableness levels. This is in line with our hypothesis that people who scored higher on these two personality traits tend to benefit more from the contact experience. There are two possible explanations. The first relates to what we hypothesized earlier - people with higher agreeableness and openness to experiences tended to be more cooperative and perspective-taking, which could improve the quality and valence of contact experiences (i.e. positive intergroup contact experiences). The other is related to ‘situational evocation and selection’. A previous study conducted by Jackson and Poulsen found that for ethnic prejudice, individuals with high agreeableness and openness tended to seek out favourable intergroup contact experiences (situational selection) and were more likely to act in a way that facilitates favourable interactions (situational evocation) [48]. In this case when contact opportunities are available, the purposive selection of favourable interactions among individuals who scored higher on agreeableness and openness ultimately lead to more positive contact experiences with the mentally ill. Although these two explanations were different, both suggest a possibility that when contact opportunities are available, individuals who scored higher on agreeableness and openness tend to have more positive contact experiences compared to their counterparts with lower scores, and such positive contacts could significantly reduce their stigma towards mental illness [47]. However, for ‘weak-not-sick’, close contact experience was a significant predictor only among those with low openness to experience, which is contradictory to findings from the other two stigma domains. This discrepancy might be caused by the fact that ‘weak-not-sick’ is more about individuals’ knowledge of mental illness which can benefit from both education and contact experiences, in contrast to the other two domains. Since the participants were all health care students who were exposed to various information on different diseases including mental illness, their knowledge depends largely on their curiosity and level of seeking and accepting new knowledge, openness to experience in other words [26, 27, 49]. Thus, health care students with higher openness would have better knowledge on mental illness and therefore lower stigmatized scores on ‘weak-not-sick’. However, better knowledge also indicated there would be less room for further improvements. This could be the reason for the non-significant correlations between contact experiences and ‘weak-not-sick’ among participants with average or high openness. It also indicated that for individuals who are less keen in acquiring new knowledge, contact experience can be a good strategy to improve individuals’ perceptions on ‘weak-not-sick’.

The results of the current study have significant implications. First of all, although personality traits (as trait-like characteristics) are quite stable, there is evidence suggesting that personality traits continue to develop, especially during young adulthood [50, 51]. In this case, future studies may explore the possibility of integrating certain personality modification interventions into the attitude campaigns especially those campaigns targeting children or younger adults. Such integration could also be applied among stigma campaigns towards clinicians, especially in their earlier stages of their career such as when they are still students. This could yield better outcomes and thus might be one of the directions for future attitude campaigns. Secondly, the moderation analyses results revealed that close contact experience is beneficial for attitudes to mental illness only among specific subgroups of health care students (i.e. individuals with lower openness or agreeableness tended to benefit less from such experiences). This could be due to the possibility that health care students with higher openness or agreeableness are more likely to seek positive contact experiences with individuals with mental illness. Although further studies are still needed to determine the exact mechanism, the current study raises the importance of individual differences in the areas of stigma and indicates that it might also be necessary to also take these differences into considerations for future stigma studies or programs. Thirdly, although prejudice and stigma are different, our study suggests that theories or models of prejudice could sometimes be applied in the area of stigma, supporting that bridging the research of these two constructs could be potentially very informative [16, 42].

The current study has several strengths. To the best of our knowledge, this is the first study in Asia which has investigated the relationship between personality and stigma towards mental illness; and it is also the first study to explore the moderating effects of personality on the relationship between contact experience and stigma. Other than extending the findings from western populations into the eastern society, the current study also identified the potential individual difference on the effects of contact experience on stigma; this will be extremely helpful for us to understand the role of personality on individuals’ attitudes towards mental illness in other contexts as well. Secondly, the study has a much larger sample size than the previous studies [20, 41]; which in turn makes the findings more robust. Lastly, a vignette-based approach was used in the current study. Compared to having the participants to rate their attitudes towards mental illness based on a general term ‘mental illness’, this approach could elicit more accurate information as ‘mental illness’ might indicate different diseases to different people.

The study findings should also be viewed with the following limitations in mind. First of all, the study sample comprised a group of health care students, which might limit the applicability of the findings to the general population. Second, the participants volunteered to participate in the current study; in this case, a self-selection bias might apply. Third, the vignette-based approach has its own limitations – for example, it is based on hypothetical scenarios, which might be different from real life occurrences. Fourthly, although it is common to use 1-item question to measure interpersonal contact in stigma research [45], this lacks test-retest reliability. Lastly, although we proposed that contact quality and valence might play an important role in the relationships of contact, personality and stigma, contact quality and valence were not measured in our study. Future studies are still needed to further explore this area.

## Conclusion

The current study confirmed the direct relationship between personality and stigma towards mental illness, with those who scored lower on openness to experience and agreeableness, and higher on extraversion, conscientiousness, and neuroticism tending to show more stigmatizing attitudes. Openness to experience and agreeableness moderated the relationship between close-contact and stigma, with individuals with average and higher levels of openness or agreeableness tending to benefit more from the close contact experience. Future studies should explore suitability of interventions based on personality traits; and test if the findings of this study can be extended to the general population.

## Additional file

Additional file 1: Vignettes used in the study (DOCX 14 kb)

## Abbreviations

ADHD: Attention deficit hyperactivity disorder
CFI: The comparative fit index
DSS: Depression stigma scale
Mini-IPIP: short form International Personality Item Pool-Five-Factor Model measure
OCD: Obsessive compulsive disorder
RMSEA: The root mean square error of approximation
RWA: Right-wing authoritarianism
SD: Standard deviation
SDO: social dominance orientation
SDS: Social distance scale
TLI: The Tucker-Lewis index
